# Micro-paper-based analytical device decorated with metal-organic frameworks for the assay of synthetic cannabinoids in oral fluids coupled to ion mobility spectrometry

**DOI:** 10.1007/s00604-023-05844-6

**Published:** 2023-06-23

**Authors:** Héctor Martínez-Pérez-Cejuela, Patricia García-Atienza, Ernesto Francisco Simó-Alfonso, José Manuel Herrero-Martínez, Sergio Armenta

**Affiliations:** Department of Analytical Chemistry, University of Valencia, Dr. Moliner 50, 46100 Burjassot, Valencia Spain

**Keywords:** Drugs, Biological samples, Paper-based devices, Solid-phase extraction, UiO-66

## Abstract

A new concept of paper-based device has been developed combining the advantages of cellulose supports and the rich surface chemistry of metal-organic frameworks (MOFs). The composite, named as NH_2_-UiO-66@paper, has been developed for the isolation of synthetic cannabinoid receptor agonists (SCRAs) in oral fluids, trying to mimic the interactions of those compounds with the human CB1R and CB2R receptors, mainly governed by hydrogen bonding and π-interactions with serine and histidine residues. MOF selection (UiO-66) and functionalization of the ligand (2-aminoterephthalic acid) has been done according to the following criteria: (i) water stability of the selected MOF, and (ii) promoting appropriate interactions with SCRAs due to the MOF nature. NH_2_-UiO-66@paper composite has been characterized in depth and the results confirmed that the material is stable at the temperature selected for thermal desorption (230 °C). Furthermore, the developed method provided appropriate precision values (RSD < 12%) and a limit of detection as low as 10 ng using ion mobility spectrometry as analytical technique. Lastly, the method has been successfully applied to the isolation of several synthetic cannabinoids from oral fluids. This method claims to be an interesting approach for expanding the combination of MOFs with sustainable support and represents a promising alternative to sophisticated and non-portable systems due to the negligible sample treatment required and the simplicity of the operation, which can be applied with screening purposes.

**Graphical abstract:**

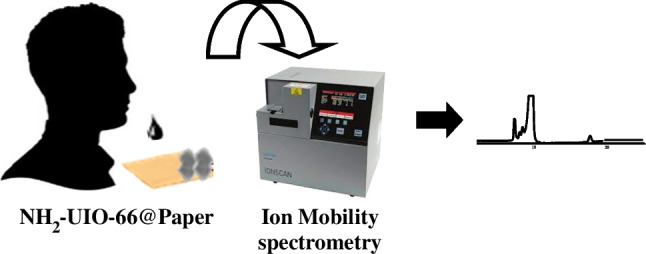

**Supplementary Information:**

The online version contains supplementary material available at 10.1007/s00604-023-05844-6.

## Introduction

Device miniaturization has attracted increasing interest as testing tool based on the portability and low cost of analytical systems, easy to use, and the possibility of rapid on-site analysis [1]. Among all the possible miniaturized platforms, paper-based materials are of special interest due to the intrinsic nature of this cellulosic support [2, 3], for instance, availability, easy derivatization, lightweight, and capillary forces without the need of external propulsion forces [4]. However, the functionalization of the cellulose surface must be performed in order to adapt its properties to the target analyte/s, combining the benefits provided by the cellulose and improved properties of functional materials [2, 3]. Up to date, different materials have been used to design paper-based composites including nanoparticles [5], graphene oxide [6], covalent-organic frameworks [7], molecularly imprinted polymers (MIPs) [8], and metal-organic frameworks (MOFs) [9]. This later group of materials is a combination of organic ligands bridged to metal centers via coordination bonds forming porous 3D networks. They have attracted the attention of many researchers due to their outstanding properties such as rich chemical surface, robustness, and great surface areas, among others [10]. The countless number of available MOFs depends on the possible combinations between different metals and organic ligands, which includes also the infinite functionalities that can be added to the structure (e.g., -NH_2_, -COOH, and -OH) pre-/post-synthesis [11–13].

In this work, we report the design and assessment of a portable and robust platform used as extraction device for the isolation of 7 different synthetic cannabinoid receptor agonists (SCRAs) in oral fluids. Considering data reported in 2022 by the UNODC Early Warning Advisory (EWA) on new psychoactive substances (NPS), a total of 1124 unique NPS have been reported to the UNODC EWA [14]. Stimulants are the largest group at 34%, followed by SCRAs with 29%. According to UNODC data, since 2009, a total of 320 different SCRAs have been reported to the UNODC EWA, being the majority of them aminoalkylindoles and aminoalkylindazoles, 13 of them identified for the first time in 2021.

At present, the drug analysis is somehow limited by the requirement of scientific laboratories equipped with highly sophisticated instruments and trained operators [15–17]. In this sense, the design of paper-based systems using Whatman#1 filter paper claims to be an alternative to face these demands. Furthermore, an active material able to retain the target compounds could increase the selectivity of the extraction method, being MOFs one of the most promising sorbent materials for analytical purposes.

However, few of the MOFs reported in the literature found practical application in bioanalysis. Selected MOFs should possess at least robustness and water stability. For this reason, the use of high-valent metal ions, such as Zr (IV), and carboxylate-based linkers to synthetize MOFs is one of the most popular methods to obtain water stable MOFs [18]. The first Zr (IV)-based MOF, UiO-66, was reported in 2008, and it is well-known for its exceptional thermal and chemical stabilities in water under a wider pH range [19]. Considering the interaction of SCRAs with human CB1R and CB2R is governed by hydrogen bonding and π-interaction with serine and histidine residues [20], the selection of MOF and functionalization of the ligand has been done trying to optimize the interaction between sorbent and analyte, although other related compounds can interact as well. Thus, NH_2_-UiO-66 using 2-aminoterephthalic acid as ligand has been proposed for paper functionalization in the extraction of SCRAs from oral fluids. In this paper-based concept, the available methods for drug monitoring are extended and it can serve as a guide for future works in order to combine MOF and cellulosic supports for their application in analytical chemistry.

## Experimental section

### Reagents and materials

All the chemicals used in the present work were of analytical grade. More information can be found in the Electronic Supplementary Material (ESM).

### Instrumentation

The detailed information of all the used equipment is shown in the ESM.

### Synthesis of NH_2_-UiO-66@paper

**Fig. 1:**
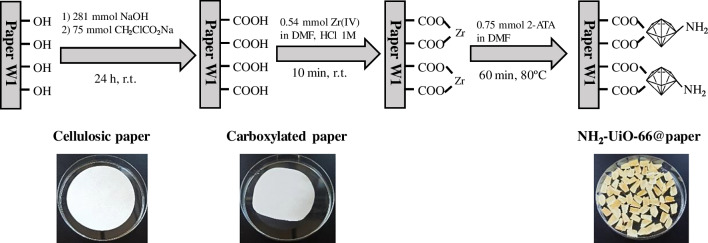
Schematic representation of the NH_2_-UiO-66@paper synthesis step by step

The preparation of the final paper-based device started with the carboxymethylation of the W1 paper [21]. The generation of MOF was accomplished following the one-pot approach. Concisely, the derivatized cellulose matrix was immersed in 20 mL of DMF containing 1 mL of conc. HCl and 0.54 mmol of ZrCl_4_. After 10 min, a solution of 0.75 mmol 2-aminoterephtalic acid in 5 mL of N,N-dimethylfomamide was added dropwise under gently stirring. The mixture was heated up to 80 °C and it was kept 1 h. The solution aspect changed from transparent to milky yellowish tonality. Carefully, the paper was taken out from the solution and rinsed several times with DMF, water, and ethanol, respectively. It is important to highlight that the paper was manipulated with tweezers and no hitting walls in order to avoid MOF losses. Finally, the material was dried over 8 h in the oven at 75 °C and carefully cut in 1 × 1 cm pieces, which were stored in a desiccator until use. Figure 1 shows a representative scheme from the entire synthesis procedure. It should be mentioned that the selected size (1 cm^2^) is due to its suitability for both the selected sample volume (100 μL) and the diameter (1.8 cm) of the thermal desorption unit of the IMS instrument.

### Analytical procedure

The SCRAs extraction from oral fluid samples were performed as follows: 100 μL of sample were carefully loaded in MOF@paper device and they were kept in contact for 4 h. Then, the extraction support was immersed in water twice in order to rinse the non-retained analytes or matrix interferents. After that, the NH_2_-UiO-66@paper was air-dried and the retained SCRAs were thermally desorbed into the IMS system. Plasmagrams were registered in positive ion mode. Nicotinamide was selected as internal calibrant (K_0_ 1.860 cm^2^ V^−1^ s^−1^). The acquisition period was fixed at 40 ms and the shutter grid width set at 0.2 ms. The counterflow of dried air (75 mL min^−1^) was introduced as drift gas at the end of the drift region. The electric field was set at 251 V cm^−1^ in the drift region with a total voltage of 1759 V and tube length 7 cm. Desorption, inlet, and drift tube temperatures were set at 230, 240, and 237 °C, respectively. The sample tray, containing the NH_2_-UiO-66@paper, was inserted in the heating zone and was kept for 30 s in that position.

## Results and discussion

### Motivation of the design

Among the vast list of MOFs developed to date, UiO-66 has been selected in this study because of its high-water stability compared to other MOF families based on their strong Zr (IV)–O bonds [22], which is a prerequisite when biological fluids will be analyzed. Furthermore, it has been previously demonstrated that indole, quinolone, and related compounds are adsorbed over pristine and functionalized UiO-66 MOFs. The adsorbed quantity of analyte increased with the number of H-acceptors and H-donors, suggesting the importance of H-bonding in the adsorption. NH_3_^+^-UiO-66 and NH_2_-UiO-66 provided the highest adsorption for both indole and quinolone [23]. Additionally, π-electron donor–acceptor interactions have been already indicated between NH_2_-UiO-66 sorbents and organic compounds [24]. The paper substrate was selected, as described in the “Introduction” section, due to the interesting characteristics that the cellulose support possesses, most of them in accordance with the sustainable principles.

Thus, considering that the most important interactions of SCRAs with human receptors are based on hydrogen bonding and π-stacking, and precisely, these interactions are present in MOF adsorption mechanisms, among others, NH_2_-UiO-66 is proposed as solid material to modify paper devices and extract SCRAs from biological fluids. This hypothesis was supported in a preliminary study, in which SCRAs were extracted using NH_2_-UiO-66 particles loaded onto SPE cartridges [25].

### Synthesis and characterization NH_2_-UiO-66@paper

**Fig. 2:**
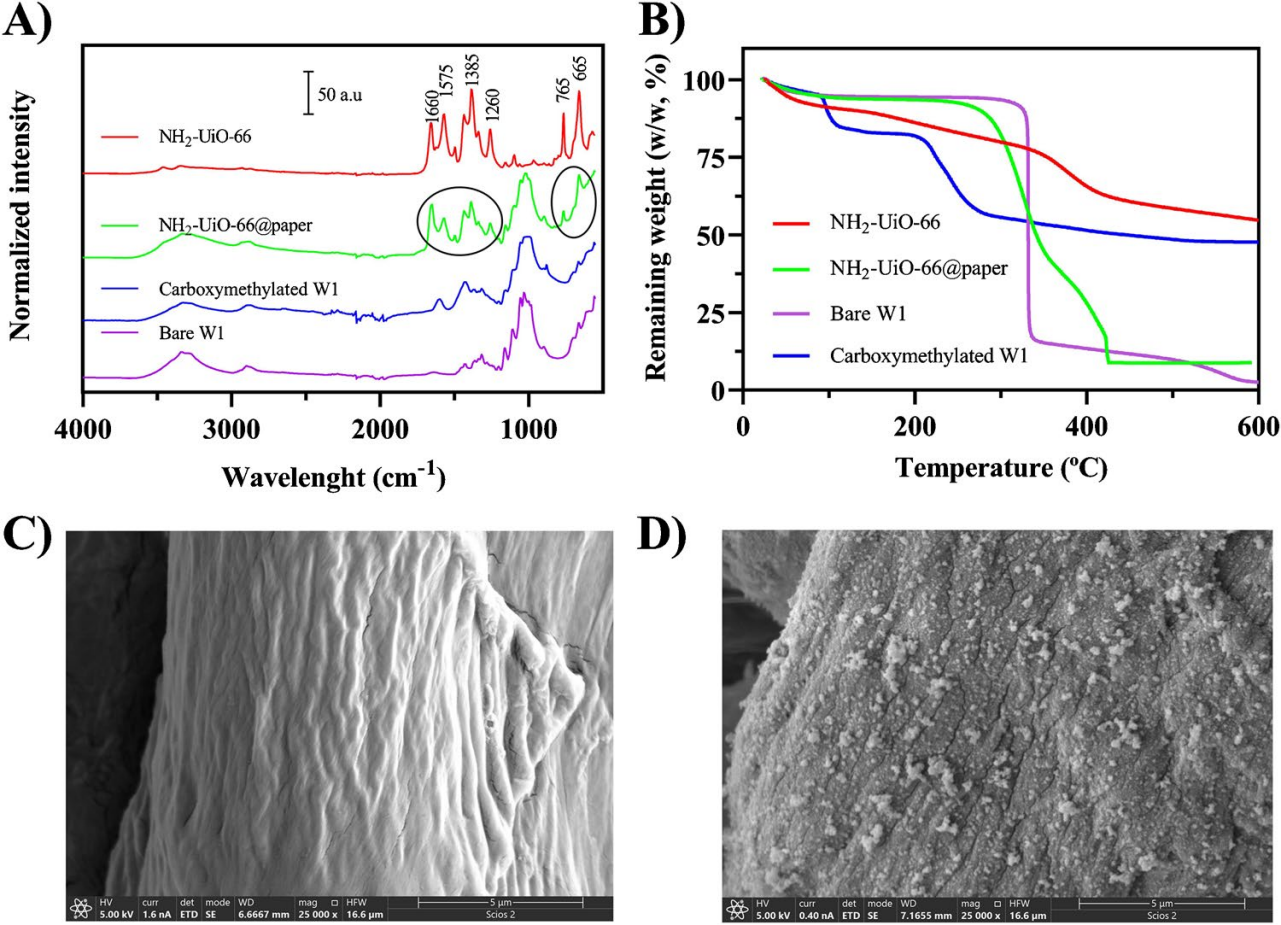
Characterization of the paper devices. **A** FTIR spectrum of NH_2_-UiO-66@paper from 4000 to 500 cm^−1^; **B** TGA analysis up to 600 °C; SEM images from **C** bare paper and **D** NH_2_-UiO-66@paper

Figure 1 summarizes the entire synthesis process. It was performed taking into account the necessity of paper surface modification to introduce anchoring groups (e.g., –COOH). In this sense, the oxidation of the hydroxyl groups, which are abundantly present in the support, was achieved using sodium chloroacetate as oxidant. After this step, the paper color did not change significantly. However, its consistency became rough, rigid, and more resistant (see image from Fig. 1). To assess a successful derivatization, carboxymethylated paper was weighed and the FTIR and TGA analyses were performed. As it can be seen in Fig. 2A, the FTIR spectrum from bare paper showed characteristic bands at 3300–3400 cm^−1^ related to O-H stretching, 2890 cm^−1^ due to CH_2_ stretching, 1425 cm^−1^ related to crystalline phase, and another at 900–1000 cm^−1^ due to amorphous phase of cellulose [26]. Furthermore, an intense band around 1050 cm^−1^ is also visible probably referred to C-O-C pyranose ring vibration [27] and C–C stretching, C–O–H, and C–C–H deformation [28]. In addition to the main characteristic bands of cellulose, carboxymethylated paper showed new characteristic absorption bands around 1600 and 1414 cm^−1^, which correspond to the anti-symmetric and symmetric stretching vibrations of COO-, respectively, which agrees with other reported works [29]. On the other hand, TGA was also performed from both materials (Fig. S2B) and the results suggest the correct process of derivatization of W1 support (more information at ESM).

Next, the in situ growth of NH_2_-UiO-66 was performed via one-pot approach. Metal ions, such as Zr (IV), can be attached to the –COOH residues generated onto the surface of the paper. In the present case, the Zr (IV) was added together with HCl (1 M) in order to slow down the hydrolysis of Zr salt and counteract the COOH deprotonation [30] and, thus, maximize the amount of bounded metal. After that, the ligand was added in order to build a homogeneous layer onto the surface. The initial white color changes to yellowish paper (see Fig. 1), when the temperature reached 80 °C (at the same time, a milky solution was being formed with the same color). Furthermore, the final paper showed a mass increase of 31 ± 5 mg (*n* = 5) with respect to the non-derivatized support. Although these notorious changes suggested a correct synthesis of the NH_2_-UiO-66@paper device, further investigation was done to support and verify this hypothesis.

FTIR spectrum of NH_2_-UiO-66@paper showed the same pattern than pristine MOF (Fig. 2 traces red and green) with lower intensities due to the lower proportion of MOF with respect to the paper. The NH_2_-UiO-66 IR spectrum, which matches with previously reported spectra [25], shows peaks around from 1700 to 1300 cm^−1^ that can be matched with C=C and O–C–O stretching vibrations. At the range 2750–3500 cm^−1^, a broad brand appears, which is presumable from O-H and N-H stretching. Furthermore, in the range 1590–1650 cm^−l^, the H-N-H bending mode is considered to predominate. The peak at 1260 cm^−1^ could be attributed to C-N stretching and, finally, the N–H wagging can be observed at 765 and 665 cm^−1^.

On the other hand, the papers before and after modification were also compared by scanning microscopy. As shown in Figs. 2C and D, the rough surface of the bare cellulose is completely decorated with MOF particles, which present low sizes and good homogeneity around the material.

Further studies to increase the MOF loading in the final device were done since MOF is the main responsible for the extraction. The synthesis conditions were varied in terms of temperature (60 and 100 °C, fixed 1 h) and time (30 and 120 min, fixed 60 °C). After the entire process, no changes were observed in the color, characterization studies neither in the analytical performance. Hence, the synthesis conditions were set at 60 °C and 1 h as convenience.

### Evaluation of thermal desorption and experimental conditions

Thermal desorption is an extraction process based on the combination of heat and a flow of inert gas, which provides a significant concentration enhancement. For this reason, several desorption temperatures were tested in the range 200–300 °C, being 230 °C the optimum value (Fig. S4). More information is available at ESM.

Furthermore, it has been previously described that the addition of solvents can improve thermal desorption processes [31] and, thus, the effect of the addition of 5 μL organic solvent on the thermal desorption of SCRAs was evaluated and the results are depicted in Fig. S5A. As it can be seen, IMS signals were doubled when 5 μL of acetonitrile or acetone were injected onto the NH_2_-UiO-66@paper before thermal desorption compared to the direct analysis of the NH_2_-UiO-66@paper.

Other important experimental parameters were optimized including sample volume and extraction time in the adsorption step. Sample volume was fixed to the maximum value that could be easily collected from volunteers. Traditionally, oral fluid volumes from 100 to 500 μL have been used in drug testing analysis [31]. However, considering the higher incidence of the dry mouth syndrome in cannabis and SCRAs users [32], and the difficulty to obtain 500 μL in heavy smokers, 100 μL was selected as most adequate sample volume. Furthermore, this volume assures the complete immersion of the paper device, and it allows an adequate interaction between sorbent and analyte during the extraction process. On the other hand, due to the neutral pH of oral fluid, with a normal range between 6.8 and 7.3 [33], and its buffer capacity, attributed to the bicarbonate concentration [34] parameters such as ionic strength or pH were not altered.

Additionally, the effect of loading time between the sample and the NH_2_-UiO-66@paper device has been evaluated from 15 min to 20 h, using MDMB-4en-PINACA (50 ng) as model compound. Figure S5B shows an increase of the MDMB-4en-PINACA signal with loading time, achieving a quantitative recovery after 4 h (<80%). This fact can be explained probably due to the low kinetics of mass transfer between the sorbent and the analytes (few micrograms of MOF are present in each device). In any case, since no stirring is needed, a high number of samples can be extracted at the same time. This fact, together with the reduced analysis time of the direct thermal desorption in the IMS procedure, can result in a high-throughput sample processing. Thus, the following parameters were selected for further experiments: (i) 100 μL sample volume, (ii) 4 h loading time, (iii) 5 μL of acetonitrile as carrying solvent, and (iv) 230 °C desorption temperature.

After this optimization, the NH_2_-UiO-66@paper was compared with the bare and carboxymethylated paper in order to check the importance of the MOF presence. None of both materials without MOF was able to retain more than 20% of MDMB-4en-PINACA, used as representative analyte.

### Analytical features of the developed method

Once the method was optimized, its suitability was assessed by studying the quality parameters. Table 1 shows the obtained results. As it can be observed, working ranges were comprised between 10 and 200 ng with linearity over 0.97 (regression coefficient) in all cases. It is also worth to mention that the LODs and LOQs achieved were as low as 1.5–3 (15–30 μg L^−1^) and 5–10 ng (50–100 μg L^−1^), respectively. These values assure the detection of SCRAs in oral fluids at previously reported concentrations, which are approximately at tens to hundreds nanograms [35]. Finally, the precision of the method was evaluated not only intra-batch (same synthesis devices) but also inter-batch (different synthesis devices). The values varied from 3 to 12% (expressed as RSD), which are good enough considering the reproducibility of IMS injection.

**Table 1:** Analytical features of the evaluated SCRAs following the proposed IMS procedure

Analyte	Working range (ng)	Linearity (*R*^2^)	LOD (ng/μg L^−1^)^1^	LOQ (ng/μg L^−1^)^1^	Precision (RSD, %)	
					Intra-batch^2^	Inter-batch^3^
JWH-081	10–200	0.981	3/30	10/100	5	8
JWH-210	7–200	0.992	2/20	7/70	4	7
THJ-2201	10–200	0.989	3/30	10/100	8	12
5F-NPB-22	5–200	0.974	1.5/15	5/50	6	9
ADB-CHMICA	10–200	0.994	3/30	10/100	5	7
MDMB-CHMZCA	10–200	0.991	3/30	10/100	8	11
MDMB-4en-PINACA	5–200	0.990	1.5/15	5/50	3	6

^1^Limit of detection (LOD) and quantification (LOQ) were calculated as three and ten times the standard deviation of a 10 ng analyte signal (100 μg L^−1^) divided by the slope of the corresponding calibration line

^2^Intra-batch values were calculated by using 6 paper devices from the same synthesis in the same day

^3^Inter-batch values were estimated by using 4 paper devices from different syntheses in different days. In the case of RSD values, a mass of analyte of 10 ng (100 μg L^−1^) was used

On the other hand, the selectivity of the NH_2_-UiO-66@paper device was evaluated using several psychoactive compounds, including amphetamine, oxazepam (benzodiazepine), and cocaine at 10 ng (100 μg L^−1^). The results indicated that the recoveries of these analytes using the optimized protocol varied between 12 and 35%, considerably lower than recovery values obtained for SCRAs (> 70%). In this sense, although the MOF capability to isolate organic compounds is not restricted to SCRA family, it is true that it works better than other psychoactive compounds. Furthermore, the use of IMS enhances the selectivity of the method due to the different migration velocities based on its mass, charge, and shape, thus, allowing the correct identification of the target analytes.

### Oral fluid analysis

The usefulness of the developed procedure was evaluated using oral fluid samples. For this purpose, several volunteers, which did not consume SCRAs, were directly analyzed, being the results under the LOD. Afterwards, those samples were spiked at two different SCRAs concentration levels and analyzed with the optimized protocol (see “Experimental section”). The results are summarized in Table 2, showing satisfactory recovery values for most of the analytes, at the different concentration levels. It should be highlighted that JWH-210 and MDMB-CHMZCA provided slightly lower results than the rest of SCRAs, from 50 to 69%. It could probably be due to the higher log *P* value of JWH-210 and MDMB-CHMZCA, 7.8 and 6.6, respectively, compared to the other evaluated SCRAs which ranges from 3.62 (MDMB-4en-PINACA) to 6.3 JWH-081. In summary, the method has demonstrated to be useful for SCRAs determination in oral fluids with any potential interferences.

**Table 2:** SCRAs recovery studies in spiked oral fluids using the developed NH_2_-UiO-66@paper protocol

Analyte (SCRAs)	Recoveries (%) ± SD (*n* = 4)			
	Sample 1		Sample 2	
	12.5 ng	125 ng	12.5 ng	125 ng
JWH-081	66 ± 5	85 ± 7	79 ± 6	100 ± 8
JWH-210	60 ± 6	61 ± 4	56 ± 4	66 ± 4
THJ-2201	100 ± 7	77 ± 5	95 ± 8	116 ± 11
5F-NPB-22	68 ± 6	72 ± 5	75 ± 6	81 ± 7
ADB-CHMICA	71 ± 6	67 ± 6	89 ± 8	109 ± 9
MDMB-CHMZCA	50 ± 3	54 ± 6	59 ± 6	69 ± 5
MDMB-4en-PINACA	77 ± 6	82 ± 6	82 ± 5	88 ± 7

It is worth to mention that this technology implies the expansion of the knowledge in the field of MOF-based materials for screening purposes. However, some limitations are still remaining in the present work such as loading time (4 h) and selectivity. The former can be faced with large batches of analyses and the latter with selective detection technique, but further studies are welcome to improve the present work.

## Method comparison

The developed MOF@paper method has been compared with some of the most recent contributions regarding the assay of synthetic cannabinoids [36–39]. Table 3 depicts some analytical parameters of these methods as well as figures of merit. In general, the developed method required lower sample volumes than those reported in the literature, being most of them biological fluids. However, the proposed method takes longer times than the rest, which can be accomplished in less than 30 min, being a parameter to optimize. It is true that some works reported evaporation steps in order to preconcentrate the sample that has not been taken into account and, obviously, with a concomitant increase of analysis time.

**Table 3:** Comparison between the developed SPE-IMs method with similar reported ones in the bibliography

Method	Material/device	Sample matrix	Sample volume required (μL)	Pretreatment time (min)	Recoveries (%)	RSD (%)	LODs (μg L^−1^)	E.F.^1^	Ref.
μ-SPE-SERS	C_18_ bed/pipette tip	Oral fluid	400	4	64	-	31	1.8	[36]
μ-SPE-LC-MS/MS	MIP/polypropylene membrane	Urine	1000	26	83–100	≤ 10	0.04–0.7	20	[37]
Online SPE-LC-MS/MS	Agilent PLRP-s/SPE cartridge	Oral fluid	-	15	33–167	≤ 20	0.4–3.8	-	[38]
SPE-LC-MS	Oasis ® HLB/6 mL SPE cartridge	Wastewater	20	1	79–136	≤ 15	0.0005–0.1	200	[39]
μ-SPE-IMS	MOF@paper/1 cm^2^ square	Oral fluid	100	240	50–116	≤ 12	15–30	33	This work

*Abbreviations: *μ-SPE*, micro solid-phase extraction; *SERS*, surface-enhanced Raman spectroscopy; *MS*, mass spectrometry; *IMS*, ion mobility spectrometry; *MIP*, molecularly imprinted polymer; *HLB*, hydrophilic-lipophilic balance

^1^Enrichment factor has been calculated taking into account the used volumes (3 μL of IMS injection volume in our case)

On the other hand, similar recoveries are obtained in all the works [36, 37, 39], except for [38], which obtained values lower than 50% and higher than 150%. Furthermore, Mulet et al. [38] also reported the highest values of RSD as precision reference. Concerning the LODs, the proposed method showed similar to these reported by SERS analysis [36], and higher than those using highly sophisticated equipment (e.g., MS detectors) [37–39]. Finally, the enrichment factor of Sánchez-González et al. [37] is comparable to MOF@paper method, whereas the preconcentration factors from Deriu’s method [36] and Pandopulos’s one [39] are significantly different. With regard to the benefits of the proposed SPE-IMS method, the method can be carried out without centrifugation/decantation step, avoiding sorbents losses. Similarly, high thermal resistance has been stated by studying the MOF@paper device at different temperatures, being an interesting option for gas desorption analysis.

## Conclusions

For the first time, we have designed and applied a composite, based on cellulose filter paper and NH_2_-UiO-66, to the isolation of synthetic cannabinoids in oral fluids. The manufacture of the NH_2_-UiO-66@paper has been performed via one-pot synthesis highlighting the simplicity and scalability of the procedure. In this sense, the procedure was followed by several characterization techniques in order to corroborate each step and assure the correct formation of the final composite. After the optimization of some key parameters such as thermal desorption and loading time, the resulting portable platform presented a satisfactory affinity towards synthetic cannabinoids, which can be retained within 4 h. The combination of both materials in a single device leads to a SCRA identification at trace scale with accessible instrumentation, which encourages to continue studying these hybrid materials from screening purposes. The true potential was also verified by applying the optimized protocol to isolation of seven SCRAs in several oral fluids from volunteers. The acceptable recoveries suggest that this tool, with cost per device lower than 10 Euro cents, can be successfully used for drug monitoring in real samples. Although this can be improved in terms of analysis time or selectivity, it would serve as guide for both researchers using MOFs as functional materials and also for researchers focused on fast in-field screening purposes.

## Supplementary Information

ESM 1

## References

[1] Hussain CM (2020) Handbook on miniaturization in analytical chemistry: application of nanotechnology. Elsevier

[2] Noviana E, Ozer T, Carrell CS (2021). Microfluidic paper-based analytical devices: from design to applications. Chem Rev.

[3] Ozer T, McMahon C, Henry CS (2020). Advances in paper-based analytical devices. Annu Rev Anal Chem.

[4] Martínez-Pérez-Cejuela H, Mesquita RBR, Couto JA (2022). Design of a microfluidic paper-based device for the quantification of phenolic compounds in wine samples. Talanta.

[5] Chen GH, Chen WY, Yen YC (2014). Detection of mercury(II) ions using colorimetric gold nanoparticles on paper-based analytical devices. Anal Chem.

[6] Morales-Narváez E, Naghdi T, Zor E, Merkoçi A (2015). Photoluminescent lateral-flow immunoassay revealed by graphene oxide: highly sensitive paper-based pathogen detection. Anal Chem.

[7] Mullangi D, Shalini S, Nandi S (2017). Super-hydrophobic covalent organic frameworks for chemical resistant coatings and hydrophobic paper and textile composites. J Mater Chem A.

[8] Wang S, Ge L, Li L (2013). Molecularly imprinted polymer grafted paper-based multi-disk micro-disk plate for chemiluminescence detection of pesticide. Biosens Bioelectron.

[9] Hassanzadeh J, Al Lawati HAJ, Al Lawati I (2019). Metal-organic framework loaded by rhodamine B as a novel chemiluminescence system for the paper-based analytical devices and its application for total phenolic content determination in food samples. Anal Chem.

[10] Yaghi OM, O’Keeffe M, Ockwig NW (2003). Reticular synthesis and the design of new materials. Nature.

[11] Ahmed I, Mondol MMH, Jung MJ (2023). MOFs with bridging or terminal hydroxo ligands: applications in adsorption, catalysis, and functionalization. Coord Chem Rev.

[12] Khan MS, Shahid M (2022) Synthesis of metal-organic frameworks (MOFs): routes to various MOF topologies, morphologies, and composites. Elsevier, pp 17–35. 10.1016/B978-0-323-90784-2.00007-1 10.1021/cr200304e 22098087

[13] Wang S, Lv Y, Yao Y (2018). Modulated synthesis of monodisperse MOF-5 crystals with tunable sizes and shapes. Inorg Chem Commun.

[14] UNODC, World Drug Report 2022 (United Nations publication, 2022) Austria. Booklet 4. Dru Market Trends. https://www.unodc.org/res/wdr2022/MS/WDR22_Booklet_4.pdf. Accessed 13 Feb 2023

[15] ElSohly MA, Gul W, Wanas AS, Radwan MM (2014). Synthetic cannabinoids: analysis and metabolites. Life Sci.

[16] Tan SN, Ge L, Wang W (2010). Paper disk on screen printed electrode for one-step sensing with an internal standard. Anal Chem.

[17] Aldlgan AA, Torrance HJ (2016). Bioanalytical methods for the determination of synthetic cannabinoids and metabolites in biological specimens. TrAC - Trends Anal Chem.

[18] Burtch NC, Jasuja H, Walton KS (2014). Water stability and adsorption in metal-organic frameworks. Chem Rev.

[19] Cavka JH, Jakobsen S, Olsbye U (2008). A new zirconium inorganic building brick forming metal organic frameworks with exceptional stability. J Am Chem Soc.

[20] Zagzoog A, Brandt AL, Black T (2021). Assessment of select synthetic cannabinoid receptor agonist bias and selectivity between the type 1 and type 2 cannabinoid receptor. Sci Rep.

[21] Heinze T, Koschella A (2005). Carboxymethyl ethers of cellulose and starch — a review. Macromol Symp.

[22] Bůžek D, Adamec S, Lang K, Demel J (2021). Metal-organic frameworks: vs. buffers: case study of UiO-66 stability. Inorg Chem Front.

[23] Sarker M, Song JY, Jeong AR (2018). Adsorptive removal of indole and quinoline from model fuel using adenine-grafted metal-organic frameworks. J Hazard Mater.

[24] Taima-Mancera I, Rocío-Bautista P, Pasán J (2018). Influence of ligand functionalization of UiO-66-based metal-organic frameworks when used as sorbents in dispersive solid-phase analytical microextraction for different aqueous organic pollutants. Molecules.

[25] Pérez-Cejuela HM, Conejero M, Amorós P (2023). Metal-organic frameworks as promising solid-phase sorbents for the isolation of third-generation synthetic cannabinoids in biological samples. Anal Chim Acta.

[26] Tabarsa T, Sheykhnazari S, Ashori A (2017). Preparation and characterization of reinforced papers using nano bacterial cellulose. Int J Biol Macromol.

[27] Laguardia L, Vassallo E, Cappitelli F (2005). Investigation of the effects of plasma treatments on biodeteriorated ancient paper. Appl Surf Sci.

[28] Proniewicz LM, Paluszkiewicz C, Wesełucha-Birczyńska A (2001). FT-IR and FT-Raman study of hydrothermally degradated cellulose. J Mol Struct.

[29] Anjali T (2012). Modification of carboxymethyl cellulose through oxidation. Carbohydr Polym.

[30] Vermoortele F, Bueken B, Le Bars G (2013). Synthesis modulation as a tool to increase the catalytic activity of metal-organic frameworks: the unique case of UiO-66(Zr). J Am Chem Soc.

[31] Drummer OH (2006). Drug testing in oral fluid. Clin Biochem Rev.

[32] Darling MR, Arendorf TM (1993). Effects of cannabis smoking on oral soft tissues. Community Dent Oral Epidemiol.

[33] Reichardt EM, Baldwin D, Osselton MD (2013). Effects of oral fluid contamination on two oral fluid testing systems. J Anal Toxicol.

[34] Almståhl A, Wikström M (2003). Electrolytes in stimulated whole saliva in individuals with hyposalivation of different origins. Arch Oral Biol.

[35] Blandino V, Wetzel J, Kim J (2017). Oral fluid vs. urine analysis to monitor synthetic cannabinoids and classic drugs recent exposure. Curr Pharm Biotechnol.

[36] Deriu C, Conticello I, Mebel AM, McCord B (2019). Micro solid phase extraction surface-enhanced Raman spectroscopy (μ-SPE/SERS) screening test for the detection of the synthetic cannabinoid JWH-018 in oral fluid. Anal Chem.

[37] Sánchez-González J, Odoardi S, Bermejo AM (2018). Development of a micro-solid-phase extraction molecularly imprinted polymer technique for synthetic cannabinoids assessment in urine followed by liquid chromatography–tandem mass spectrometry. J Chromatogr A.

[38] Mulet CT, Tarifa A, DeCaprio AP (2020). Comprehensive analysis of synthetic cannabinoids and metabolites in oral fluid by online solid-phase extraction coupled to liquid chromatography-triple quadrupole-mass spectrometry. Anal Bioanal Chem.

[39] Pandopulos AJ, Bade R, O’Brien JW (2020). Towards an efficient method for the extraction and analysis of cannabinoids in wastewater. Talanta.

