# Supplementary material for: Micro-paper-based analytical device decorated with metal-organic frameworks for the assay of synthetic cannabinoids in oral fluids coupled to ion mobility spectrometry
Source: Mikrochim Acta. 2023 Jun 23;190(7):271. doi: 10.1007/s00604-023-05844-6 (PMC10289984; doi:10.1007/s00604-023-05844-6)
Supplement: Supplementary file 1 — ESM 1 [file 604_2023_5844_MOESM1_ESM.docx]

**Electronic Supporting Material on the Microchimica Acta publication entitled**

**Micro-paper-based analytical device decorated with metal-organic frameworks for the assay of synthetic cannabinoids in oral fluids coupled to ion mobility spectrometry**

**Héctor Martínez-Pérez-Cejuela*, Patricia García-Atienza, Ernesto Francisco Simó-Alfonso, José Manuel Herrero-Martínez, Sergio Armenta***

**Department of Analytical Chemistry, University of Valencia, C/Dr. Moliner, 50, 46100 Burjassot, Valencia, Spain**

**Table of contents**

**Page S1-S2.** Experimental Section

**Page S2-S5.** Results and Discussion

**Page S6.** Physico-chemical parameters of SCRAs (Table S1)

**Pages S7.** EDX and mapping analyses of paper (Figure S1)

**Pages S7.** EDX and mapping analyses of NH_2_-UiO@paper (Figure S2)

**Pages S8.** SEM images (Figure S3)

**Page S9.** SCRAs desorption temperature studies (Figure S4)

**Pages S10.** Method optimization, solvent nature and temperature (Figure S5)

**Page S11.** References

**Experimental Section**

*Reagents and materials*

Zirconium tetrachloride, 2-aminoterephthalic acid, N,N-dimethylfomamide (DMF), methanol, ethanol, sodium hydroxide, sodium chloroacetate, Whatmann #1 (W1) papers were all purchased from Merck-Sigma-Aldrich. Other reagents and organic solvents (e.g. chloroform, acetonitrile, etc.) were from analytical grade unless otherwise stated.

The SCRAs (JWH-081, JWH-210, MDMB-4en-PINACA, 5F-NPB-22, ADB-CHMICA, THJ-2201, MDMB-CHMZCA) were obtained from Sigma-Aldrich (San Louis, USA), LGC standards (Barcelona, Spain) and “Drug Control Laboratory from Valencian Community Government” (Valencia, Spain). Name, common abbreviation, molecular weight and chemical structure of the different analyzed SCRAs are summarized in Table S1. Individual stock solution at 1000 mg L^-1^ were prepared and stored at -20 ºC. Daily mixture solutions at 1 mg L^-1^ were used.

Milli-Q water (Millipore, Bedford, MA, USA) with a resistivity lower than 18 MΩ cm^-1^ was used.

*Instrumentation*

Analyte detection was performed using an IONSCAN-LS IMS from Smiths Detection (Morristown, NJ, USA), equipped with a 63Ni foil radioactive ionization source and IM station software (V5.389). For material characterization, scanning electron microscopy with focused ion beam (SEM-FIB) micrographs were acquired with the microscope model SCIOS 2 (ThermoFisher Scientific), which was also coupled to an energy dispersive X-ray analyser (EDAX). A transmission electron microscope (TEM) coupled to a digital camera AMT RX80 model JEM-1010 JEOL (Akishima, Japan) was employed. Powder X-ray Diffraction (p-XRD) spectra were obtained by using a D8 Advance A25 diffractometer (Bruker). Attenuated total reflection Fourier-transform infrared (FTIR) spectra were acquired on a Bruker spectrometer (Bremen, Germany) model Tensor 27, equipped with a 9 reflection diamond/ZnSe plate and DuraSamplIR II accessories (Smiths Detection Inc., Warrington, UK).

*Paper carboxymethylation*

For paper derivatization, a filter paper W1 was immersed (diameter of 70 mm) in a solution of 3.75 M sodium hydroxide for 1 min. Then, 2.9 g of sodium chloroacetate were added (final conc. 1 M) and the mixture was homogenized. The piece of paper was kept in this solution for 24 h at atmospheric conditions. After this period, the modified paper was rinsed three times with ethanol and water, respectively. Next, the carboxymethylated paper was dried at 75 ºC for 8 h.

**Results and Discussion**

*Characterization of NH_2_-UiO-66@paper*

TGA was performed from both bare MOF (red trace), bare paper (purple trace), carboxymehylated paper (purple trace) and NH_2_-UiO-66@paper (red trace) (Figure 2B) First, the bare paper is completely destroyed around 350 ºC, which is expected due to the organic nature of the support. In contrast, the carboxymethyl cellulosic paper presented an increased thermal stability [1], being the first weight loss at 75 ºC (moisture) and a second big loss at 200 ºC. This later material was able to maintain up to 50 % (w/w) of the initial mass over 600 ºC.

On the other hand, the TGA depicts a different behaviour between the carboxymethylated paper and the NH_2_@UiO-66, which can be explained due to the presence of the MOF particles. The red trace, representing the pristine powdered NH_2_-UiO-66, shows no significant weight loss until 400 ºC, indicating the thermostability of this material. In this sense, our hypothesis revolves around the effective coating of the surface with the MOF particles by using the several active sites previously created in the carboxymethylation step. Hence, the many coordination bonds between Zr(IV) metal nodes and the –COOH residues allows the complete covering of the paper. The subsequent growth of the NH_2_-UiO-66 confers great stability to the final composite not only in terms of thermal resistance, but also towards different solvents used in this work.

Prompted by the need of stablishing an amount of MOFs onto the surface, a single device (1x1 cm) underwent EDX-ray and elemental analyses. The former (Figure S1 and S2) indicates that the Zr content is ca. 3 % wt. (n= 10), and the elemental analysis suggests a total content of N in the final device around 0.07 % wt. On the other hand, EDX from NH_2_-UiO-66 powder indicates a Zr amount of ca. 30 % and 6% of N, which is in accordance with theoretical calculations from the molecular formula (C_48_H_34_N_6_O_32_Zr_6_) 31% and 5 % for Zr and N, respectively. As it can be easily calculated, the proportion between MOF and paper can be estimated at 1/100 mass ratio. Therefore, one paper device, which weighs approximately 10.5-11.3 mg (n=25), should contain around 100 µg of NH_2_-UiO-66.

*Evaluation of thermal desorption*

Thermal desorption is an extraction process based on the combination of heat and a flow of inert gas, which provides a significant concentration enhancement. It becomes a good alternative to solvent desorption because of their main advantages including increased sensitivity (since the whole sample is analyzed) and reduction of organic solvents consumption in the extraction step. However, the solid sorbent used for analyte preconcentration should be thermally stable at the high temperatures employed in the desorption process.

Desorption temperature should be fixed as high as possible to quantitatively desorb the analytes without compromising the thermal stability of the material. Thus, further complementary studies to TGA were carried out in order to in-depth explore the thermal stability of the NH_2_-UiO-66@paper using MDMB-4en-PINACA as model compound. The effect of the desorption temperatures on the analyte signals and NH_2_-UiO-66@paper integrity was evaluated in the range between 200 and 285 ºC. The obtained results were depicted in Figure S4. The selection of these temperatures has been done considering the previous TGA results and the maximum temperature of the thermal desorber of the IMS instrument, which is 300 ºC. As it can be observed in Figure S$A, the higher desorption temperature, the darker the filter becomes. This fact can be matched with its TGA profile (trace green), where it can be clearly observed that at temperatures lower than 230 ºC, the material remains stable. These findings ares also supported by the digital photographs, where the colour change is appreciable at temperatures higher than 250 ºC, and with SEM micrographs (Figure S3), with a partial degradation of the MOF coating. Delve into this study, the registered data from the amount of analyte desorbed is observed in the plot of Figure S4A. The first interesting thing to observe is the good precision obtained by the system, which assures the feasibility of this study. Figure S4B showed the IMS signal obtained after the analysis of the NH_2_-UiO-66@paper at different desorption temperatures. As it can be seen, the signal of MDMB-4en-PINACA can be observed at 17.7 ms with a mobility constant value (K_0_) of 1.0024  cm^2^ V^-1^ s^-1^. It can be observed that, in all the cases, MDMB-4en-PINACA appears isolated. However, when desorption temperatures increased, especially at higher temperatures than 250 ºC, a massive number of signals appeared from 6 to 15 ms. Those signals were probably due to partial MOF decomposition that could result in the release of the ligand, 2-aminoterephthalic acid, and related compounds. Although, the IMS signals of MOF thermal degradation products did not overlap with that of MDMB-4en-PINACA, competitive ionization mechanisms could be observed by charge competition in the ionization region with the target molecules. For all of these facts, the desorption temperature was set at 230 ºC for further studies. It has been previously described that addition of solvents can improve thermal desorption processes from PTFE swabs [2]. It is based on the preconcentration effect occurred when a liquid droplet containing analyte/s contacts a solid surface with a temperature much higher than the boiling temperature of the liquid. At the last moment of liquid evaporation, those analytes are desorbed from the tiny droplet with assistance from explosive solvent evaporation. Thus, the effect of the addition of 5 µL of different solvents on the thermal desorption of SCRAs from NH_2_-UiO-66@paper has been evaluated using MDMB-4en-PINACA (50 ng) as model compound. In all the cases, no analyte peak was observed during the smooth solvent evaporation process and MDMB-4en-PINACA appeared as a quite sharp peak, at 17.7 ms desorption time, probably corresponding to the explosive solvent evaporation event. In this sense, the enrichment factor can be estimated as 33-fold (100 µL of loading volume divided 3 µL of assistant solvent) multiplied by recoveries values (77% in average), giving values around 25 times.

As it can be seen in Figure S5A, IMS signals were doubled when 5 µL of acetonitrile or acetone were injected onto the NH_2_-UiO-66@paper before thermal desorption compared to the direct analysis of the NH_2_-UiO-66@paper.

**Table S1.** Physico-chemical parameters and molecular structures of the studied analytes.

| **Analytes** | **Molecular structure** | **Chemical formula** | **Log P_O/W_^a^** | **Mw**  **(g mol^-1^)** |
| --- | --- | --- | --- | --- |
| JWH-081 |  | C_25_H_25_NO_2_ | - | 371.46 |
| JWH-210 |  | C_26_H_27_NO | 7.5 | 369.5 |
| MDMB-4en-PINACA | 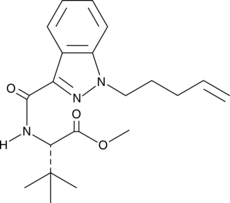 | C_20_H_27_N_3_O_3_ | 4.3 | 357.4 |
| 5F-NPB-22 | 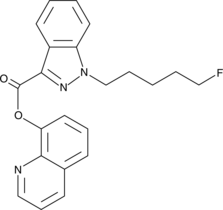 | C_22_H_20_FN_3_O_2_ | 4.9 | 377.4 |
| ADB-CHMICACA | 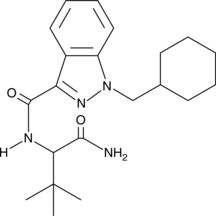 | C_21_H_30_N_4_O_2_ | 4.2 | 370.5 |
| THJ-2201 | 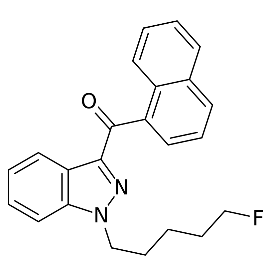 | C_23_H_21_FN_2_O | 5.8 | 360.4 |
| MDMB-CHMZCA | 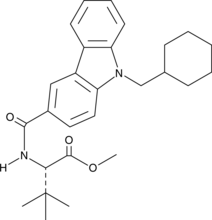 | C_23_H_32_N_2_O_3_ | 6.6 | 434.6 |

**Figure S1.** EDX and mapping analysis of paper substrate.

**Figure S2.** EDX and mapping analysis of NH_2_-UiO-66@paper device.

**Figure S3.** SEM micrograhs of A) bare MOF, NH_2_-UiO-66; b) NH_2_-UiO-66 collected from the generated dispersion from the paper device synthesis; c) after heating at 230 ºC for 30 s; d) after heating at 280 ºC for 30 s.

**Figure S4.** SCRAs desorption temperature studies from NH_2_-UiO-66@paper after the retention at different temperatures. A) IMs signal for the target compound and B) plasgrams from said study. Model compound: MDMB-4en-PINACA. Analyte amount: 20 ng. Desorption time: 30 s. Other experimental conditions are detailed in Experimental Section.


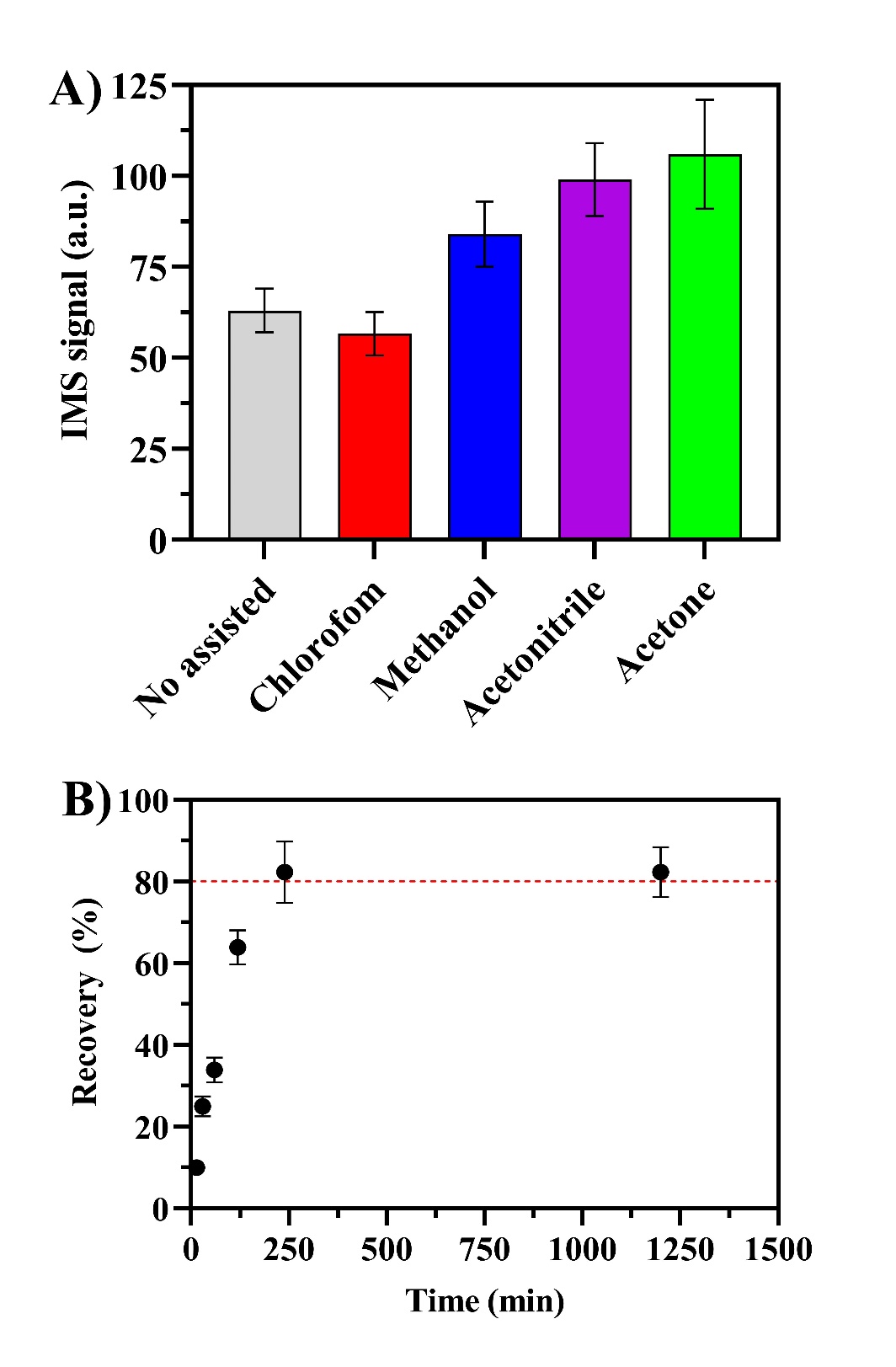


**Figure S5.** Method optimization: A) Nature of assisted-solvent for thermal desorption process and B) Extraction time. Both experiments were performed using 100 µL of MDMB-4en-PINACA at 250 µg L^-1^. Other experimental conditions can be checked at Materials and Methods Section.

**References**

1. Anjali T (2012) Modification of carboxymethyl cellulose through oxidation. Carbohydr Polym 87:457–460. https://doi.org/10.1016/j.carbpol.2011.08.005

2. Wang W, Xu C, Ruan H, et al (2020) Solvent assisted thermal desorption for the on-site detection of illegal drugs by a miniature ion trap mass spectrometer. Anal Methods 12:264–271. https://doi.org/10.1039/c9ay02202c
